# Comprehensive quality control utilizing the prehybridization third-dye image leads to accurate gene expression measurements by cDNA microarrays

**DOI:** 10.1186/1471-2105-7-378

**Published:** 2006-08-14

**Authors:** Xujing Wang, Shuang Jia, Lisa Meyer, Bixia Xiang, Li-Yen Chen, Nan Jiang, Carol Moreno, Howard J Jacob, Soumitra Ghosh, Martin J Hessner

**Affiliations:** 1 The Max McGee National Research Center for Juvenile Diabetes, Department of Pediatrics, The Medical College of Wisconsin and Children's Hospital of Wisconsin, 8701 Watertown Plank Road, Milwaukee, WI 53226, USA; 2 The Human and Molecular Genetics Center, The Medical College of Wisconsin, 8701 Watertown Plank Road, Milwaukee, WI 53226, USA; 3 NimbleGen Systems Inc., 1 Science Court, Madison, WI 53711, USA

## Abstract

**Background:**

Gene expression profiling using microarrays has become an important genetic tool. Spotted arrays prepared in academic labs have the advantage of low cost and high design and content flexibility, but are often limited by their susceptibility to quality control (QC) issues. Previously, we have reported a novel 3-color microarray technology that enabled array fabrication QC. In this report we further investigated its advantage in spot-level data QC.

**Results:**

We found that inadequate amount of bound probes available for hybridization led to significant, gene-specific compression in ratio measurements, increased data variability, and printing pin dependent heterogeneities. The impact of such problems can be captured through the definition of quality scores, and efficiently controlled through quality-dependent filtering and normalization. We compared gene expression measurements derived using our data processing pipeline with the known input ratios of spiked in control clones, and with the measurements by quantitative real time RT-PCR. In each case, highly linear relationships (R^2^>0.94) were observed, with modest compression in the microarray measurements (correction factor<1.17).

**Conclusion:**

Our microarray analytical and technical advancements enabled a better dissection of the sources of data variability and hence a more efficient QC. With that highly accurate gene expression measurements can be achieved using the cDNA microarray technology.

## Background

Microarray technology allows a comprehensive examination of gene expression profiles and the regulations of their changes at a whole genome level [1-3]. It has great potential in the study of complex human diseases [4]. However, the technology is prone to noise and low reproducibility [5]. Correlations with other platforms including RT-PCR [4,5], and between different microarray platforms are often unsatisfactory [6-9]. On the other hand, many disease processes involve subtle gene perturbations that require highly accurate gene expression measurements. The noise in microarrays if not adequately reduced, can obscure the true biological variations and presents an obstacle for data-mining tools to distinguish biology from artifacts. For this reason rigorous QC standards are needed for the microarrays [10]. This in turn requires a clear understanding of the sources of data variability, so that the contributing factors can be appropriately dissected and most efficiently controlled.

Because of the lack of consistent quality control (QC) standards, spotted arrays fabricated in academic laboratories are usually more susceptible to QC problems than commercial arrays [6-8]. Their advantages include much reduced fabrication cost and higher content flexibility than commercial arrays. For example, they can be designed to target specific genetic pathways, or to perform promoter analysis for genes of interest [11]. Therefore developing a generalizable, efficient microarray QC scheme for spotted arrays is important.

We have previously reported a microarray image processing software *Matarray*, which specializes in quantitative QC of data acquisition [12,13]. Using it we have shown that several major sources of data variability are readily identifiable from the post-hybridization image, including high or non-uniform noise profiles, low or saturated signal intensities, and irregular spot sizes and shapes. Their resultant effect on data reliability can be well characterized through the definition of a set of individual quality scores each measuring the impact of a corresponding factor, and a composite score *q*_*com*_, which gives an overall assessment of the data quality acquired from each spot on the array [12]. Through numerous experiments we have demonstrated the advantages of utilizing the ratio-*q*_*com *_plot for data filtering and normalization [12,13]. Nevertheless, there are sources of variability that cannot be directly or quantitatively evaluated from the post-hybridization image. One important example is the quality of array fabrication. The generation of microarray slides involves coating of the glass slides, printing up to tens of thousands of amplified cDNA or oligonucleotide "probes" and fixing/blocking of the slide. During this process, variable amounts of material can be deposited and/or retained on the activated glass surface depending a number of variables. When the amount of immobilized probe is inadequate the measurements made on such arrays can be unreliable [14-18]. Noise and artifacts introduced to the arrays at this stage will also directly affect the quality of hybridization. Until recently, such problems have been difficult to quantitatively evaluate and control for each and every array, since the array is typically "invisible" prior to hybridization [14,16,17]. To overcome this difficulty, we have made a significant technological advancement in microarray QC by conceiving and developing a three-color microarray platform [15-17], which we termed third dye array visualization (TDAV) technology [19]. The approach labels the cDNA probes printed on the array slides with a cyanine dye-compatible third dye (TD) fluorescein [16], and makes prehybridization quantitative assessment of array quality possible, so that precious samples as well as laboratory and analytical efforts will not be wasted over poor-quality slides. In the last several years, the quantitative third dye threshold for slide QC has been extensively investigated by us [15-17].

In this report, we further investigate the advantage of TDAV in better dissecting the sources for data variability at the spot level, and develop a data filtering and normalization procedure that incorporates the information from the TD image. We utilize data from four different microarray experiments to validate our procedure. We evaluate the accuracy of our microarray measurements by comparing them with the known input ratios for spiked in control clones, and with the measurements by quantitative real time RT-PCR.

## Results and discussion

### TD intensity and gene-dependent bias in ratio measurements

**Figure 1:**
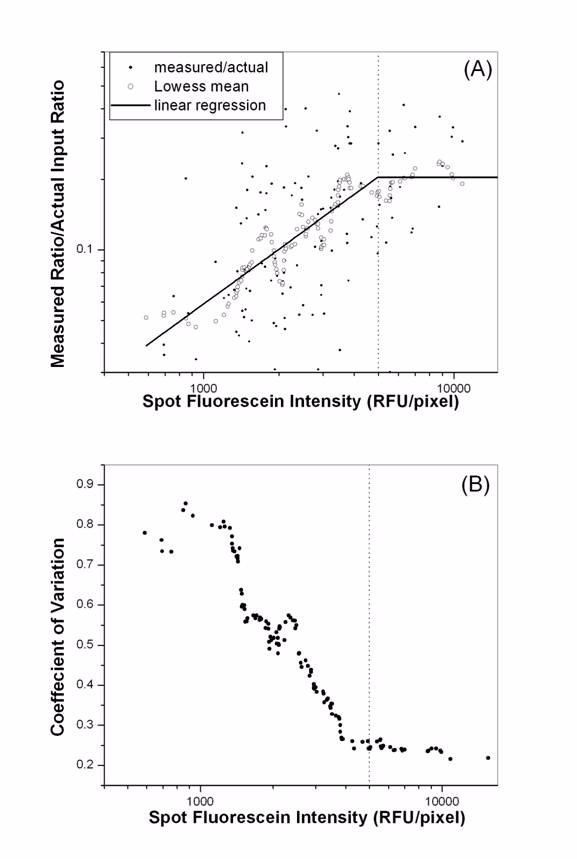
Increased data compression and variability with decreasing spot TD intensity. Data presented are from the *Arabidopsis *clone spiked in at a ratio of 30:1. (A) Significant further data compression occurred when spot TD intensity falls below 5,000 RFU/pixel. '•' are individual data points, 'o' represent the LOWESS mean of data, and the straight-line is the linear regression. (B) CV in ratio measurements was presented against spot TD intensity. Data variability increased significantly when spot TD intensity falls below 5,000 RFU/pixel.

Compression in microarray measurements (measured ratio is less than the actual ratio) have often been observed [5,20]. Furthermore, the compression may depend on the ratio or the intensity levels [5,20] leading to gene-specific biases, which are difficult to calibrate. The exact cause of the compression is still not clear, and its characteristics have not been well quantified. Our previous studies have indicated that the amount of immobilized probes on an array available for hybridization can affect the fidelity of the hybridization [16,17]. Therefore, we have examined this question in relation to spot TD intensity utilizing the spiked-in *Arabidopsis *clones from the rat thymus experiment (experiment 1). Details of the design of this experiment are described in the Methods. Spots either saturated or possessing high background were eliminated using *Matarray *[12]. After filtering, 896 (out of the total 1216) data points were available for analysis. Figure 1 gives the result for transcripts spiked in at 30:1 (Cy5:Cy3). In a perfect measurement, the ratio of measured fold of change versus actual should be "1". Indeed, compression is observed through the whole spectrum of TD intensity (figure 1A). Moreover, the compression is not constant. When the TD spot intensity falls below ~5,000 RFU/pixel, the data compression and data variability dramatically increases. This is consistent with our previous studies which suggested that under our array scanning standard, only arrays with mean signal levels > 5,000 RFU/pixel were able to generate reliable hybridization data [16,17]. In order to quantify this relationship further, we have calculated the mean behavior of the compression using LOWESS [21], and fit the LOWESS mean with a piecewise function consisting of two linear segments joined together by a short quadratic function (solid lines in figure 1A). The quadratic function ensures that non-linear least squares optimization can be used [22]. We find that indeed above the threshold value of 5,000 RFU/pixel the compression is constant; whilst below this value the compression is increasingly more severe with decreasing TD intensity (slope = 0.78, with R^2^~0.82, p < 0.0001). A similar trend exists for other input ratios and the results are summarized in table 1. These results indicate: (1) the degree of compression both above and below the TD intensity threshold is ratio dependent, the higher the fold-of-change is, the more compression the data will exhibit. (2) Significant further compression in ratio measurements below the TD intensity threshold occurs for all input ratios. (3) There is a dye bias in the characteristics of data compression.

We have also examined the data variability in relation to the TD spot intensity. At each spiked in ratio, we determined the standard deviation (SD) and the coefficient of variation (CV) in ratio measurements for every data point using its 20 nearest neighbors on the ratio-TD intensity plot. We find that above 5,000 RFU/pixel both SD and CV are essentially constants. With deficient TD intensities below 5,000 RFU/pixel SD increases initially followed by a drop at very low TD intensities due to the severe data compression in ratio measurements, whilst CV increases monotonically with decreasing TD intensity. The CV results for the data points corresponding to the spiked in ratio of 30:1 are given in figure 1B. In a real experiment where the transcript abundance for different genes spans a wide range and their folds of change vary, gene-dependent artifacts in measurements will occur. These results revealed that inadequate probe amount is an important major source of data variability that could cause complex features in data compression.

### TD intensity and the spatial-dependent bias

**Figure 2:**
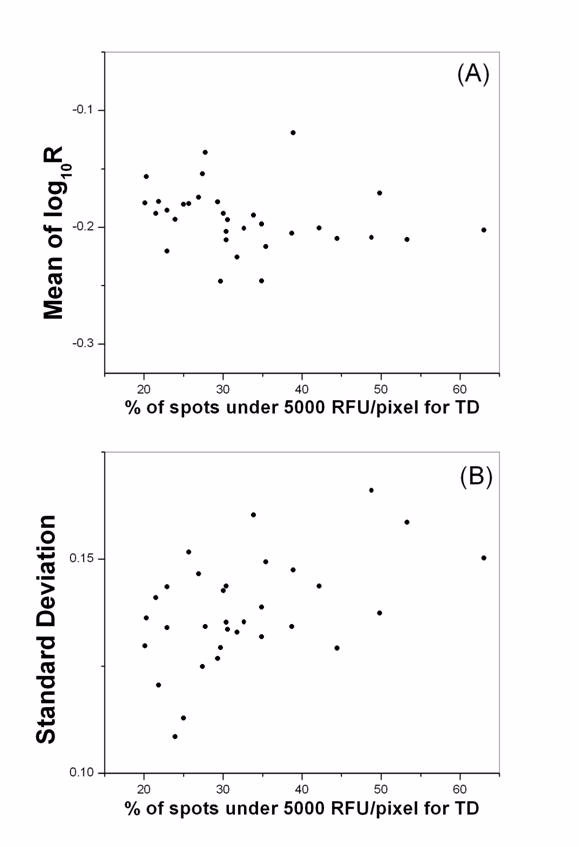
The disparity in the amount of probe printed is a major source of pin difference in microarrays. Using one hybridization from the rat thymus experiment, we calculated the mean (A) and the SD (B) in log ratio for data under each pin, and plotted them against the percentage of spots with TD intensity below 5,000 RFU/pixel. There is a clear increase of SD when there are more poor-quality spots under the corresponding pin.

Sources of variation often have localized characteristics across the whole slide. One major type of such spatial-dependent bias is the heterogeneity in the printing characteristics among the pins. Its exact cause is not clear and its characteristics not well characterized. Normalization methods have been designed to correct spatial bias. For example, the local mean normalization [23], and the pin-dependent localized intensity LOWESS normalization [24]. However they could lead to spurious results when the proportion of differentially expressed genes is high [25]. Efficient normalization requires proper dissection of the causes for bias and minimization procedures designed accordingly. We have investigated the pin issue using the BB rat thymus data. For each of the 32 pins, we determined the mean and SD of the ratio measurements, and correlated the results with the number of spots that fell below the TD threshold intensity of 5,000 RFU/pixel. We found that when there were few poor-quality spots for all pins, they did not show significant difference. Most of our arrays (>95%) that have passed our pre-hybridization QC [16,17] are in this category. However, when the amount of failed spots exceeded 20%, a positive correlation between SD in ratio measurements and the percentage of failed spots became evident. Figure 2 illustrated a typical result from one such hybridization. Whilst the mean in log ratio distribution did not change much from pin to pin, clearly data variability increased (nonlinearly) with more spots that failed the TD intensity threshold cutoff. When the amount of failed spots is very large for most pins, exceeding 50%, the pattern gets more complicated because of the severe data compression (see figure 1). In contrast, no obvious correlation was observed between pin heterogeneity and cyanine intensities (data not shown). The results here indicate that the amount of material each pin deposits is a major cause for pin difference, and hence it can be better controlled through our TDAV technology.

### Incorporating TD information in data filtering and normalization

Results in the preceding sections suggest that the TD intensity is a major factor that causes spot-level data reliability. In addition, other artifacts on the TD image can also influence the accuracy of expression measurements from post-hybridization images, including noise, spot size and shape irregularities [16,17]. Based on these observations we formulated a quality measure for every spot from the TD image by defining

*q*_TD _= *q*_int _*q_com, TD _    (1)

where *q*_com, TD _is the composite TD image quality score, defined according to signal-to-noise ratio, spot size, and background levels and variation, similarly as given in the equation (7) of [12]. *q*_int _is given by:

qint⁡={1,intensity≥thresholdintensity/threshold,intensity<threshold     (2)
 MathType@MTEF@5@5@+=feaafiart1ev1aaatCvAUfKttLearuWrP9MDH5MBPbIqV92AaeXatLxBI9gBaebbnrfifHhDYfgasaacH8akY=wiFfYdH8Gipec8Eeeu0xXdbba9frFj0=OqFfea0dXdd9vqai=hGuQ8kuc9pgc9s8qqaq=dirpe0xb9q8qiLsFr0=vr0=vr0dc8meaabaqaciaacaGaaeqabaqabeGadaaakeaacqWGXbqCdaWgaaWcbaGagiyAaKMaeiOBa4MaeiiDaqhabeaakiabg2da9maaceqabaqbaeaabiGaaaqaaiabigdaXiabcYcaSaqaaiabbMgaPjabb6gaUjabbsha0jabbwgaLjabb6gaUjabbohaZjabbMgaPjabbsha0jabbMha5jabgwMiZkabbsha0jabbIgaOjabbkhaYjabbwgaLjabbohaZjabbIgaOjabb+gaVjabbYgaSjabbsgaKbqaaiabbMgaPjabb6gaUjabbsha0jabbwgaLjabb6gaUjabbohaZjabbMgaPjabbsha0jabbMha5jabc+caViabbsha0jabbIgaOjabbkhaYjabbwgaLjabbohaZjabbIgaOjabb+gaVjabbYgaSjabbsgaKjabbYcaSaqaaiabbMgaPjabb6gaUjabbsha0jabbwgaLjabb6gaUjabbohaZjabbMgaPjabbsha0jabbMha5Hqaciab=Xda8iabbsha0jabbIgaOjabbkhaYjabbwgaLjabbohaZjabbIgaOjabb+gaVjabbYgaSjabbsgaKbaaaiaawUhaaiaaxMaacaWLjaWaaeWaceaacqaIYaGmaiaawIcacaGLPaaaaaa@8983@

In *Matarray *the default threshold = 5,000 RFU/pixel.

**Figure 3:**
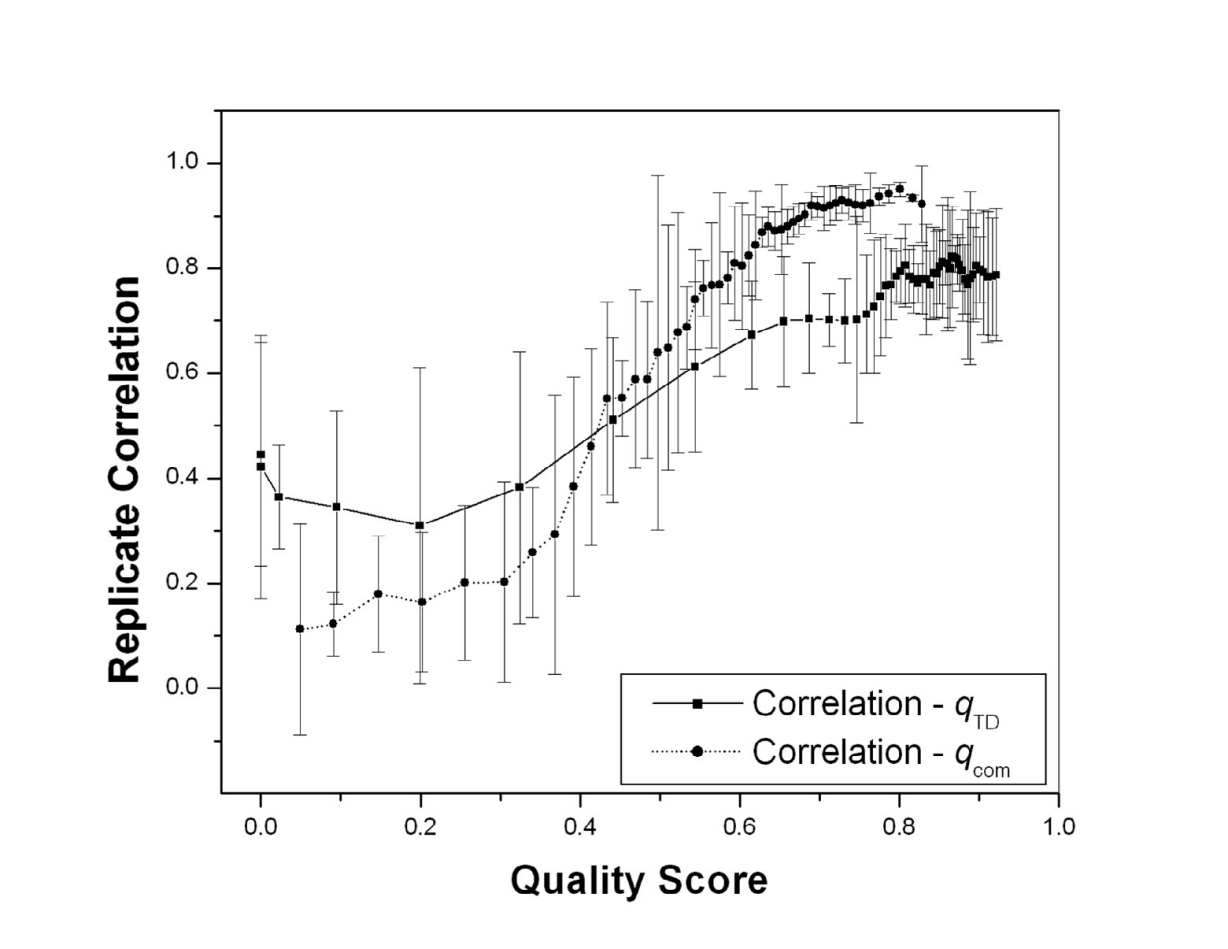
Data filtering utilizing quality scores. The correlation coefficients (mean and SD) between the 6 direct replicate hybridization pairs in data set 4 is plotted against the quality scores *q*_TD _and *q*_com_, showing that filtering by either will improve the replicate consistency.

Utilizing data from the four microarray experiments we examined the replicate consistency, and the agreement between microarray measurements and the known input ratios for the spiked in *Arabidopsis *clones, with respect to *q*_TD_. We found that like *q*_com _(which was defined for the hybridized cyanine images [12]), *q*_TD _captures well the data variability with higher *q*_TD _spots yielding less variation. For example, for each pair of direct replicate hybridizations in this study, we obtained the genes that exhibit differential expression (DE) at p = 0.05. We divided them into 50 bins, and for genes in each bin we determined the mean *q*_TD _and *q*_com_, and the Pearson correlation in log ratio measurements between the replicates. A typical result is given in figure 3. Filtering by either *q*_TD _or *q*_com _leads to significant improvement in replicate consistency. Notice that majority of the spots have high *q*_TD _due to the fact that all the slides we use for hybridization have already been pre-selected using TDAV [16,17]. We have also found that there is no significant correlation between *q*_TD _and *q*_com _(R<0.5), which validates that they are two non-redundant quality measures each capturing a different major source of data stability, and QC by each is necessary.

**Figure 4:**
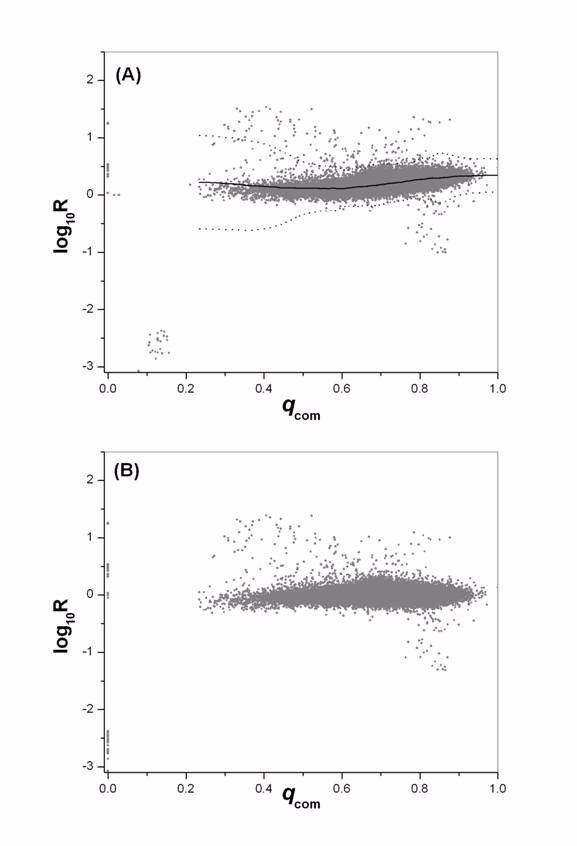
Quality dependent data filtering and normalization. Data are from one hybridization between day 65 and day 40 DP rats of experiment 1. (A) Log ratio distribution before normalization is plotted against *q*_*com*_. Spots with *q*_*com *_< 0.20 exhibit significantly increased variability, and will be reset to *q*_*com *_= 0. Normalization will be performed for all spots with *q*_*com *_> 0.20. Also shown are the normalization factor (solid line) and the local 3 SD from mean (dotted line). (B) The same data after filtering and normalization.

Incorporating our new quality score definition for the TD image, we have developed the following filtering and normalization procedure:

1. Evaluate the log *R *- *q*_TD _plot and decide on a filtering threshold for *q*_TD _. Normally this will be the value where there is an abrupt increase in data variability, in the same fashion as shown in figure 4A (which described the log*R *- *q*_com _filtering and normalization). The quality score for all spots below this threshold will be reset to *q*_TD _= 0.

2. Perform a local *q*_TD _-dependent normalization for all data points with *q*_TD _> 0 utilizing the robust scatter plot smoother LOWESS [13,21,24].

3. Evaluate the log *R *- *q*_com _plot and decide on a filtering threshold for *q*_com _. The quality score for all spots below this threshold will be reset to *q*_com _= 0, as described in figure 4A.

4. Perform a local *q*_*com *_-dependent LOWESS normalization for all data points with *q*_com _> 0. The LOWESS fit for SD will also be determined, and the Z-score will be calculated for normalized log *R *every spot by: Z=normalized log⁡Rlocal SD
 MathType@MTEF@5@5@+=feaafiart1ev1aaatCvAUfKttLearuWrP9MDH5MBPbIqV92AaeXatLxBI9gBaebbnrfifHhDYfgasaacH8akY=wiFfYdH8Gipec8Eeeu0xXdbba9frFj0=OqFfea0dXdd9vqai=hGuQ8kuc9pgc9s8qqaq=dirpe0xb9q8qiLsFr0=vr0=vr0dc8meaabaqaciaacaGaaeqabaqabeGadaaakeaacqWGAbGwcqGH9aqpdaWcaaqaaiabb6gaUjabb+gaVjabbkhaYjabb2gaTjabbggaHjabbYgaSjabbMgaPjabbQha6jabbwgaLjabbsgaKjabbccaGiGbcYgaSjabc+gaVjabcEgaNjabdkfasbqaaiabbYgaSjabb+gaVjabbogaJjabbggaHjabbYgaSjabbccaGiabbofatjabbseaebaaaaa@4C7D@[13]. Figure 4B gives the log ratio after normalization for the same data set given in figure 4A.

After all normalization a final quality score will be defined

*Q*_f _= *q*_*TD*_•*q*_*com *_    (3)

Only spots with *Q*_f _> 0 will be retained for further data mining and modeling.

### The efficiency of our filtering and normalization procedure

**Figure 5:**
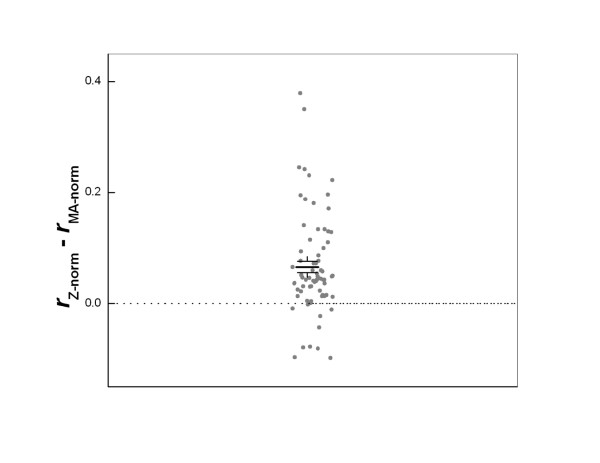
Comparison of Z-norm and MA-norm methods. Data shown are the differences in the correlation coefficients *r *between all 74 direct replicate pairs from the four data sets using the two normalization approaches. X-axis values are random numbers assigned to each data point in order to separate them. For most cases, Z-norm leads to better replicate correlations. The solid lines show the mean difference and the standard error of the mean. The difference is significant with p < 0.0001.

The integration of our technical and analytical developments has resulted in a unique procedure for comprehensive, quantitative microarray data processing and QC. To demonstrate the advantage of our filtering and normalization method, we compared it to a commonly used localized LOWESS normalization method that utilizes the ratio-intensity dependence [24] (often termed MA-LOWESS normalization). Data from all four experiments were processed both by our pipeline (termed Z-norm) and the MA-LOWESS (termed MA-norm) procedure. All spots with *Q*_f _= 0 on any replicate slide were dropped (~10% of all spots). We calculated the correlation coefficient between replicate hybridizations for common DE genes at p= 0.05 according to both normalization procedures. There were totaling 74 pairs of replicate hybridizations and good agreements were observed, with mean replicate correlation *r *= 0.73 ± 0.21 according to Z-norm. The difference between the two methods are presented in figure 5, revealing a better (P < 0.0001) overall performance by our processing pipeline. The improvement by our method is small (the mean difference in *r *is 0.06) due to the already high data quality.

### The accuracy of gene expression measurements

**Figure 6:**
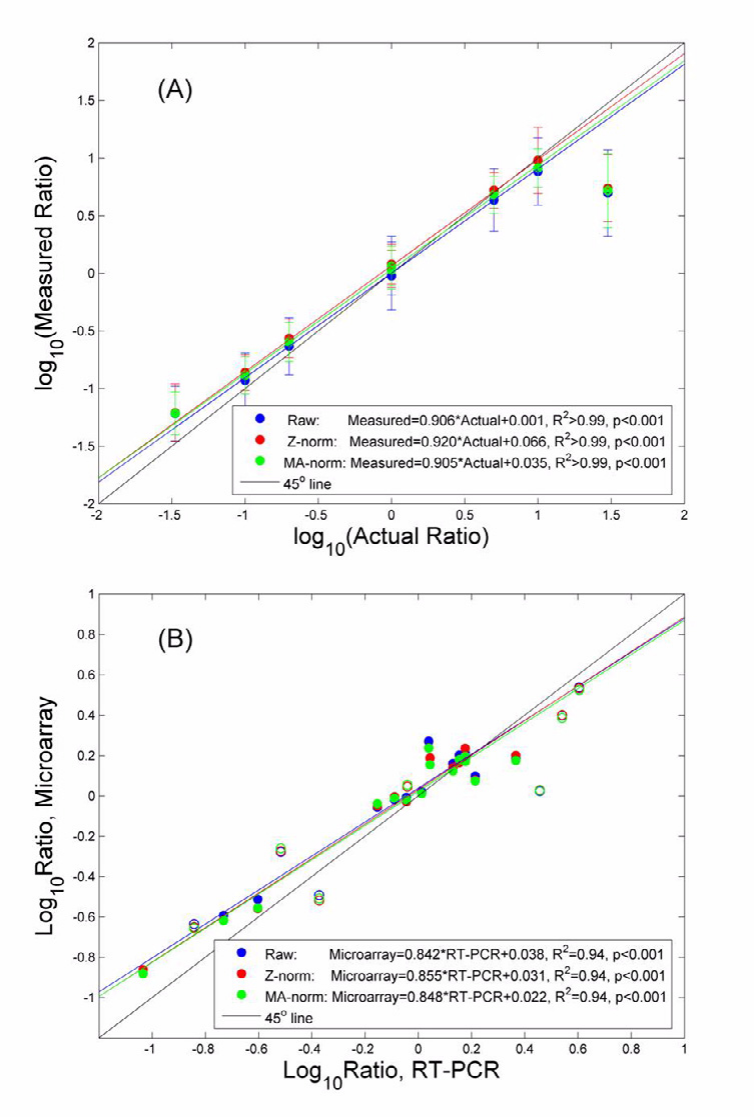
Accuracy of gene expression measurements by our microarray platform. (A) The measured ratio is compared with the actual ratio, using raw ratio measurements (Raw), ratio after our normalization pipeline (Z-norm), and ratio after MA-LOWESS normalization (MA-norm). Good agreements between measured and actual are observed. The last data point is excluded from the linear regressions. (B) Measurements by microarray are compared with those by RT-PCR in the rat liver experiment. Again a highly linear relationship is observed, with very small compression in the microarray measurements. Our method exhibits a moderate, insignificant improvement over MA-norm. Seven genes with poor quality microarray data (open circles) were excluded from the linear regression.

*Arabidopsis *clones that were spiked at known ratios in experiment 1 were utilized to assess the accuracy of our microarray measurements. A highly linear relationship between the measured and the actual ratios was observed (figure 6A), with the exception of the last data point (of spiked-in ratio 30:1, Cy5:Cy3). There are two possible causes for the discrepancy at this data point: (1) intensity saturation in one channel can lead to under-estimation of the fold difference between the two dye channels. A close look of the intensity profiles for the spots contributing to this data point revealed that the saturation is insignificant (<10% pixels for all spots) as we have only included spots with *Q*_f _> 0 in the analysis [12]. (2) Ratio measurements can have significant compression at a high fold of change, as we have demonstrated in table 1. We believe this is the major cause for the non-linearity at the last data point. Excluding it all normalization procedures led to linear regressions with *R*2 >0.99, *p *< 0.001 over a dynamic range of ~300 fold. Overall the measured data exhibited a moderate compression over the actual, with the slope of the linear regression always less than 1. Z-norm led to a small, insignificant improvement over MA-norm. Again this is likely due to the fact that all arrays used in our experiments were pre-selected using TADV [16,17] and the data were already of high quality.

In figure 6B the microarray measurements are compared to the RT-PCR results for the 22 genes in the rat liver experiments, and an overall good agreement is observed. The RT-PCR platform is generally considered more quantitative and accurate than the microarrays [5]. 7 (open circles) of the 22 genes were identified as poor-quality data points as their *Q*_f _= 0 on at least one array. Excluding these genes a highly linear relationship (*R*^2 ^~ 0.94, *p *< 0.001) existed for the remaining 15. If we were to calibrate microarray measurements using the RT-PCR results as a standard, we would have *R*_corrected _= (*R*_measured_)^*q*^, with a correction factor q~10.855=1.17
 MathType@MTEF@5@5@+=feaafiart1ev1aaatCvAUfKttLearuWrP9MDH5MBPbIqV92AaeXatLxBI9gBaebbnrfifHhDYfgasaacH8akY=wiFfYdH8Gipec8Eeeu0xXdbba9frFj0=OqFfea0dXdd9vqai=hGuQ8kuc9pgc9s8qqaq=dirpe0xb9q8qiLsFr0=vr0=vr0dc8meaabaqaciaacaGaaeqabaqabeGadaaakeaacqWGXbqCcqGG+bGFdaWcaaqaaiabigdaXaqaaiabicdaWiabc6caUiabiIda4iabiwda1iabiwda1aaacqGH9aqpcqaIXaqmcqGGUaGlcqaIXaqmcqaI3aWnaaa@3A21@. This is much better than a *q *~1.88 previously reported [26]. If the 7 poor-quality data points were to be included, the agreement between the two platforms drops to *R*^2 ^~ 0.90 with a correction factor *q *~ 1.27. This result strongly indicates that with stringent, efficient QC protocols, cDNA microarrays are able to generate high quality, quantitative gene expression measurements comparable to that by RT-PCR.

### The impact on the biology

How much impact on the biological interpretation can such improvement in data quality bring about? To answer this question we have performed ontological analysis for the DE genes in each experiments using OntoExpress [27] and EASE [28]. We found no significant difference in the pure number of DE gene predictions between data processed by either Z-norm or MA-norm (p > 0.5). However, at ontology level, a general trend appeared suggestive of Z-norm being able to lead to more focused, local biological themes, usually with more significant p-values (data not shown). For example, in experiment 3 at 6 hr after drug treatment, the apoptosis progression has been established with at least 40% cells were apoptotic according to the Annexin V/PI double staining [29]. EASE analysis of the DE genes after Z-norm predicted enhanced presence of genes belonging to regulation of cell cycle, cell proliferation/death, lysosome, lytic vacuole, nucleus, etc, most of which were closely related to apoptosis. Using DE genes predicted after MA-norm, about one third of these categories were not detected. The interpretation of ontological analysis is a complex issue, with many unresolved problems [30]. For example, since most of the ontological categories are not independent, it is still an open question on how to best recap the findings and evaluate significance [30]. Therefore a quantitative evaluation of our findings awaits further methodology development in the field of ontological analysis.

## Conclusion

In this report we have shown that when the probe amount is inadequate, severe compression in gene expression measurements occur with complex, gene-specific characteristics. Likewise, the normal variation in the amount of probes printed and immobilized is a major source for printing pin-dependent artifacts in the data. Utilizing TDAV, these problems rooted from array fabrication can be effectively dissected from hybridization problems, and efficiently controlled through the definition of a quality score *q*_TD_. In addition, we have developed a comprehensive two-step data filtering and normalization procedure based on the log *R *- *q*_TD _and log *R *- *q*_com _plots, which was found to be more efficient than the commonly used MA-LOWESS approach. By confirming our microarray data with the known input ratio of spiked in controls clones, and with RT-PCR, we demonstrated that acquiring accurate measurements using cDNA microarrays is achievable with our TDAV technology and our data QC procedure. Furthermore, in a recent study where we compared measurements from our cDNA microarrays with those from Affymetrix and Agilent oligonucleotide array platforms, we observed a high correlation among the three, with no significant differences in terms of data quality. Specifically, using ANOVA we have found that the different platforms did not cause more variation than the replicate hybridizations within each individual platform [31]. This is in contrast to the recent reports of poor performance by academic arrays which did not use our scheme [6-8]. Thus we wish to emphasize that academic labs can still perform high quality, inexpensive microarray experiments with our platform.

Our algorithms, although initially developed for cDNA arrays, are potentially applicable to spotted oligonucleotide arrays as well. Recently we have developed the three-color oligonucleotide array platform by introducing a third-dye labeled universal tracking oligonucleotide into the printing buffer, thus the quality of array fabrication can be quantitatively evaluated through the measurements of the tracking oligonucleotide [32]. A high quality microarray platform will allow lab investigators to focus on their biological questions instead of the technical issues of the data, and will allow statisticians and bioinformatics investigators to develop more powerful complex analysis approaches.

## Methods

### Microarray slide fabrication

All the microarrays used in this report were fabricated in house with TDAV technology using an OmniGrid arrayer equipped with a stealth print head with 32 SMP3 MicroQuill pins (Telechem International, Sunnyvale, CA). The University of Iowa rat library, consisting of 35,040 clones, was used as a source of probe for the cDNA microarrays, and was printed over two poly-L-lysine coated glass slides, each possessing 18,432 features. Control clones were also printed on each array including β-actin, GAPDH, and 9 *Arabidopsis *clones, as well as negative controls including PolyA and spotting buffer. Specifically, the *Arabidopsis *clones were printed in a 2 fold serial dilution from 200 ng/ul to 6.25 ng/ul (6 dilutions in each series), 4 replicate series on each slide. To ensure consistent prehybridization TD image collection, we have implemented a confocal laser scanner calibration method utilizing FluorIS (CLONDIAG, Jena, Germany), a non-bleaching, reusable, calibration/standardization tool [16]. This enabled us to set up universal, quantitative QC rules according to TD signal information. All the arrays used in our microarray experiments have passed our QC as previously described [16,17].

### Microarray data

All microarray data analyses were performed using our in-house *Matarray *package [12,13]. Information from the prehybridization TD image as well as the two post hybridization cyanine images were acquired. Data from four different microarray experiments are utilized in this report. They comprise: (1) Profiling of BioBreeding (BB) rat thymus. Gene expressions were compared between the thymus of diabetes prone DR*lyp/lyp *(referred to as DP in this report) and diabetic resistant DR+/+ (DR) rats at day 40, and between day 65 and day 40 DP rats. This analysis utilized 4 animal pairs for each comparison, and 4 replicate hybridizations were performed for each pair, with 2 hybridizations reverse labeled to control for dye bias, totaling 32 hybridizations. For the 16 hybridizations (8 each way of dye labeling) that compared day 65 and day 40 DP rats, the labeling reactions of total thymus RNA were spiked with 4 *Arabidopsis in vitro *transcripts (cellulose synthase, chlorophyll a/b binding protein, ribulose-1,5-bisphosphate and triosphosphate isomerase) at known input ratios (30:1, 10:1, 1:1, and 1:0, respectively), giving rise to a total of 1216 data points. These clones enabled an evaluation of microarray measurement accuracy through the comparison of measured output ratios to the known RNA input ratios. (2) Gene expression profiling of the kidney from an end-stage renal failure (ESRD) rat model. Three pairs of 22 week-old Fawn Hooded Hypertensive (FHH) rats and control August Copenhagen Irish (ACI) rats were compared. For each animal pair, 2 replicate hybridizations were performed, with 1 reverse labeled, totaling 6 hybridizations. (3) Time course profiling of apoptosis progression in pancreatic islet β cells. Cells from a rat β cell line RIN-m5F were treated with a protein kinase C inhibitor staurosporine [33] at a high dose of 1 μM, and a low dose of 1 nM for 2, 4, and 6 hours, and were compared for differential gene expressions. At each time point, 6 replicate hybridizations were performed, with 3 of them reverse labeled, totaling 18 hybridizations. Cell apoptosis status were confirmed using Annexin V/PI double staining method as described in [29]. (4) Profiling and comparison of liver gene expressions from day 65 BB-DR and Wistar-Furth (WF) rats. In this experiment, 4 animals from each strain were sacrificed and equal amounts of purified total RNA from the animals of the same strain were pooled. The two pools were then compared in a total of 6 replicate hybridizations, with 3 of them reverse labeled.

### Real time quantitative RT-PCR of rat liver samples

In the last experiment that profiled the BB-DR and WF rat liver, expression of 22 genes that were deemed of biological interest according to our previous experiments had also been measured by quantitative real time RT-PCR. Two sets of primers were designed for each locus with Oligo 6.66 (Molecular Biology Insights, Cascade, CO) and were blasted against the whole genome to ensure gene specificity. The primers were synthesized by Sigma Genosys (The Woodlands, TX). A Rotor-Gene 3000 (Corbett Research, Morelake, Australia), was used to conduct monoplex quantitative real-time RT-PCR as previously described [34]. Relative gene expression data (fold of change) between samples was determined using the mathematical model described in [35].

## Abbreviations

TDAV, third dye array visualization; QC, quality control; TD, third dye; SD, standard deviation; CV coefficient of variation; LOWESS, locally weighted scatter plot smoothing; DE, differentially expressed.

## Authors' contributions

XW, MJH, SG, and HJJ designed research; XW, SJ and MJH, performed research; LM, CM, BX performed experiments; CM, LYC, BX and HJJ contributed reagents/analytical tools; SJ, NJ, LYC, XW analyzed data; XW, MJH, SG wrote the paper.

## Figures and Tables

**Table 1:** Compression in microarray measurements. The compression depends on the fold-of-change, and exhibits complex characteristics when the spot TD intensity falls below 5,000 RFU/pixel. Data presented are from the spiked-in *Arabidopsis *in vitro transcripts in experiment1. At each input ratio, the mean compression factor (ratio between the measured and input ratio) is determined for all spots with TD intensity above 5,000 RFU/pixel, and a linear regression is performed between the compression and the spot TD intensity for all spots below 5,000 RFU/pixel, in the same fashion as described in figure 1

Input ratio (Cy5:Cy3)	Compression factor for spots > 5,000 RFU/pixel	Slope of linear regression (< 5,000 RFU/pixel)
30:1	0.18	0.78 (R^2^~0.82, p < 0.0001)
10:1	0.77	0.42 (R^2^~0.62, p < 0.0001)
5:1	0.87	0.27 (R^2^~0.46, p < 0.0001)
1:1	1.05	0.10 (R^2^~0.30, p < 0.03)
1:5	0.86	0.05 (R^2^~0.04, p < 0.06)
1:10	0.84	0.14 (R^2^~0.18, p < 0.0001)
1:30	0.55	0.27 (R^2^~0.77, p < 0.0001)
